# *An Account of the Poisonous Effects of the Seeds of the Datura Stramonium* Linn

**Published:** 1794

**Authors:** James Johnson

**Affiliations:** Surgeon at Lancaster.


VIII. *An Account of the poifonous Effects of the Seeds of the Datura Stramonium* Linn. *By Mr.* James Johnfon, *Surgeon at Lancafter.*

ON the 12th of November, 1789, about five o'clock in the afternoon, Mifs S. of this town, aged twenty years, fwallowed fome of the feeds of the ftramonium or thorn-apple. Three hours afterwards fhe began to be fick, and her mother gave her a mixture of flour of muftard and water to make her vomit, and it produced this effect feveral times. At eleven o'clock the fame fame evening I vifited her, and immediately fufpected what fhe had been eating from the defcription her mother gave me of the plant, (the patient having deftroyed all fhe had of it); and the next day I was convinced of its being the datura ftramonium, by a frefh fpecimen of the plant, which was procured from the fame place where the other had been gathered.

As fhe had vomited feveral times before I was called to her, I contented myfelf with adminiftering a purgative medicine, which procured feveral ftools. She paffed, however, a reftlefs night, complained of great pain of her ftomach and left arm, fancied fhe faw objects that did not exift, and had repeatedly a fenfation of a flafhing light, which made her think that fhe faw it lighten.

After the operation of the purgative, fhe took repeatedly of an oily emulfion.

Nov. 14, As her ficknefs, and pain of her ftomach, ftill continued, I gave her an emetic, of antim. tartar. and ipecacuanha, which brought off feveral of the feeds of the ftramonium.

15. The ficknefs and uneafinefs at her ftomach ftill continuing, fhe repeated the purgative medicine, and it operated feveral times.

 16. She

16. She had paffed a much better night, and was free both of ficknefs and of the pain at her ftomach.

17. As fhe now had no complaint but weaknefs, fhe was directed to take the Peruvian bark infufed in port wine; and when I faw her again on the 20th, fhe found herfelf quite recovered.

Of the inftances of the poifonous effects of ftramonium, recorded by different writers, thofe defcribed by Dr. Rufh *, Profeffor Lobftein †, and Dr. Fowler, feem to have the greateft affinity to the cafe I have related.

The fubject of Dr. Rufh's account was a girl between three and four years old. She had fever, delirium, tremor of her limbs, and a general eruption on her fkin. The real caufe of thefe fymptoms not being fufpected, purgative medicines, the warm bath, and cataplafms to the feet were employed, but without effect. At length it was difcovered that fhe had fwal-

* An Account of the Effects of the Stramonium or Thorn Apple. By Benjamin Rufh, M. D. Profeffor of Chemiftry in the College of Philadelphia.—See Tranfactions of the American Philofophical Society, Vol. I. p. 318.

† In an Appendix to a Differtation de Vegetabilibus Venenatis Alfatiæ, by F. A. Guerin. 4to. Strafburgh, 1766.

lowed lowed ſome of the ſeeds of the ſtramonium. She was now vomited with emetic tartar, but brought up only phlegm. Sweet oil, however, mixed with a little caſtor oil, brought away a great number of the ſeeds by ſtool. The relief this oily medicine gave her, occaſioned it to be repeated every day for a week; but the tremor ſtill continued, at times, in her hands, and ſhe was blind and ſtupid. The pupils of her eyes were much dilated, and ſhe caught at the bed-clothes, and every thing around her, like a perſon in the laſt ſtage of a fever.

Being perſuaded that the oily mixture had evacuated all the ſeeds that were in the inteſtines, Dr. Ruſh ſuſpected that her complaints were kept up by a few ſeeds retained in the ſtomach. He therefore gave her a larger doſe of emetic tartar, which brought up eighty of the ſeeds; but the ſtupor and blindneſs ſtill continuing, the emetic was again repeated, and brought up above twenty more of the ſeeds; after which all her complaints vaniſhed.

Dr. Ruſh ſuppoſes, that in this caſe the effects of the poiſon would have been greater, if the ſeeds had been freſher; for they were, it ſeems, of the preceding year's growth, and were become dry and hard.

He adds, in a note, that Dr. Bond and Dr. Harris had informed him, that in a ſimilar caſe they had experienced good effects from lemon juice, after the ſtrongeſt vomits had failed to relieve the patient.

The caſe related by Dr. Lobſtein was as follows:

Two children at Straſburgh, a brother and a ſiſter, the former ſix, the latter nine years old, ate ſome ſeeds of the ſtramonium about five o'clock, P. M.

At nine the girl complained of laſſitude, and went to bed. Her mother ſoon after finding her feveriſh, and talking in her ſleep, concluded that ſhe was about to have ſome eruptive diſeaſe, and gave her ſomething warm to drink. She continued in this ſtate till one o'clock in the morning, when the boy alſo, who had ſlept in another room with his father, was become extremely reſtleſs. At ſeven, A. M. when Dr. Lobſtein firſt ſaw theſe children, they were both much convulſed; their faces and cheeks were ſwelled; their lips were of a deep red; their eye-lids ſwelled and cloſed: on ſeparating the eye-lids, the pupils of the eyes were found to be much dilated; the eyes themſelves were convulſed. Their bellies were tumid, but not hard.

hard. When they were loudly ſpoken to, or when any body touched them, their convulſions became more frequent and violent. In each of them the tongue was ſwelled and protruded out of the mouth; and their fauces were ſo conſtricted that reſpiration was performed with difficulty. By degrees the ſpaſms became leſs violent, and their ſkin, from being intenſely hot, became moiſt; their pulſe was ſtill quick, but ſofter. They now rejected every kind of liquid, and ſeemed to labour under hydrophobia; for on offering a cupful of drink to them, Dr. Lobſtein found that the moment it touched their lips the ſpaſms returned with great violence.

While he was endeavouring to aſcertain the cauſe of theſe alarming ſymptoms, he accidentally obſerved, in the chamber, a portion of the plant which had occaſioned the miſchief. He now began, therefore, to excite vomiting; but no ſeeds of the ſtramonium were rejected. He was more ſucceſsful, however, with clyſters, which were repeatedly adminiſtered, to the number of twelve, and brought away ſeeds of the plant with the ſtools. The bellies of the patients now became leſs tumid; their reſpiration was eaſier; and the ſwelling of their faces fubfided. Lemon juice, and likewife vinegar and water, were now adminiftered to them.

At eleven, A. M. when Dr. Lobftein faw them a fecond time, he found the girl more quiet, but ftill at times convulfed. She drank freely of vinegar and water; her pulfe was fofter; fhe perfpired confiderably; the tumefaction of her belly had fubfided; and fhe was free from delirium, but the pupils of her eyes were ftill dilated. The boy remained unrelieved.

At eight, P. M. the girl was fufficiently recovered to give her friends an account of the plant, the feeds of which fhe and her brother had eaten. She had got fome refrefhing fleep, but her pupils were ftill fo dilated as not to contract on the approach of a candle.

The boy likewife now began to mend; and at eleven, P. M. opened his eyes and fpoke to thofe about him. The next day they were both of them pretty well recovered.

Of thefe cafes, from Dr. Lobftein, I have been induced to be the more particular in my account, as the Differtation to which they are annexed is fcarce; but, for the particulars of

Dr.

Dr. Fowler's cafes, I fhall content myfelf with referring to the collection in which they are publifhed, as being a work pretty generally in the hands of medical readers in this country*.

From none of the facts hitherto publifhed, relative to the poifonous effects of this plant, does it feem to be afcertained what quantity of the feeds may be fwallowed without deftroying life. Their effects, no doubt, will vary according to the foil and climate in which the plant is produced, and according to the greater or lefs degree of maturity to which the capfules have arrived.

In one of the two cafes related by Dr. Fowler, the patient, a girl not quite fix years old, is faid to have fwallowed three-fourths of the feeds of a frefh, ripe, middle-fized thorn-apple; and I have reafon to think that my patient fwallowed nearly as many feeds as were contained in one apple; but it would feem that in the Eaft Indies thefe feeds are much more deleterious; for there, according to Dr. An-

* See Edin. Med. Comment. Vol. V. page 161.—References to other inftances of the poifonous effects of the feeds of the plant in queftion, may be found in the late Profeffor Murray's Apparatus Medicaminum, 8vo. Gotting. 1776. Tom. I. p. 456, and in Dr. Woodville's Medical Botany, Vol. II. p. 338.

derfon *, the feeds of one thorn-apple are generally fufficient to induce immediate death.

Both my cafe and Dr. Rufh's fhow the utility of repeated and active emetics in fuch cafes, and how long the fymptoms may be kept up by the retention of feeds in the ftomach. In the cafe of my patient, as we have feen, feveral of the feeds were brought up on the fecond day, by means of an antimonial emetic, notwithftanding the patient had been fo freely puked on the day fhe fwallowed the feeds; and in Dr. Rufh's patient a fimilar effect refulted from a large dofe of emetic tartar, at the end of a week, notwithftanding an emetic of lefs activity, given foon after the poifon had been fwallowed, brought up only phlegm. All the cafes I have fpoken of tend to fhow that our endeavours in fimilar inftances fhould be directed to the fpeedy evacuation of the feeds, by means of active and repeated emetics, and of purgative medicines and clyfters.

This plant, though not originally a native of Europe, is now but too common in every part of it. Our countryman Gerarde fpeaks of it as being, in his time, a rare and ftrange plant

* London Med. Journal, Vol. X. p. 285.

in in England*; but it is now ſo frequent, that by later writers we find it deſcribed among our indigenous plants.

* See Johnſon's edition of Gerarde's Herball, Folio, London, 1633, p. 348.

